# 

**Published:** 1788

**Authors:** 


### CONTENTS.

Page

I. *ADDITIONAL Observations on Amputation. By Mr.* James Lucas, *one of the Surgeons of the General Infirmary at Leeds, and Member of the Corporation of Surgeons in London.* — — 223

II. *A Case of Hydrophobia. By Mr.* James Russel, *Apothecary in London.* — 256

III. *An Account of the successful Termination of a Case attended with Symptoms of Phthisis Pulmonalis; with Remarks on the Treatment of that Disease. By* William May, *M. D. Physician at Truro in Cornwall.* 268

IV. *A singular Case of Diabetes, consisting entirely in the Quality of the Urine; with an Inquiry into the different Theories of that Disease. By* Thomas Cawley, *M. D. late chief Surgeon to the Forces in Jamaica.* - 286

V. *Observations on Pemphigus. By* Stephen Dickson, *M. D. Fellow of the College of Physicians, and one of the King's Professors of Physic in the City of Dublin, M. R. I. A.* &c. — *From The Transactions of the Royal Irish Academy,* — — 309

CATALOGUE *of* BOOKS, - 325

